# Newly qualified midwives’ experiences of caring for women in the maternity setting: An integrative review

**DOI:** 10.18332/ejm/169667

**Published:** 2023-09-20

**Authors:** Jeanette Gauci, Rita Pace Parascandalo

**Affiliations:** 1 Mater Dei Hospital, Ministry of Health, Msida, Malta; 2 Department of Midwifery, Faculty of Health Sciences, University of Malta, Msida, Malta

**Keywords:** newly qualified midwives, new graduate midwives, junior midwives, newly registered midwives, beinner midwives, early career midwives

## Abstract

**INTRODUCTION:**

This integrative review aims to explore the experiences of newly qualified midwives (NQMs) when caring for women in the maternity setting.

**METHODS:**

An integrative review (IR) method was chosen based on a systematic approach using Cooper’s 1984 framework for transparency and rigorousness. A total of 2428 articles were located, duplicate records were removed, leaving 1428 records. All titles and abstracts were reviewed and 91 articles were then retrieved in full text. After applying inclusion and exclusion criteria, 22 studies were ultimately included in this IR. Appraisal tools were used for each study included in this review according to its research approach.

**RESULTS:**

Literature demonstrated how NQMs’ wellbeing is at stake after registration as they go through a tumultuous period full of psychological stress, fear and other surges of negative emotions. The importance for NQMs to form new and trusting relationships with colleagues and women patients is highly evident in the literature.

**CONCLUSIONS:**

Transitioning from a student to a midwife brings about stress and tension especially when NQMs take full responsibility for the women under their care, knowing that their decisions might have a direct impact on the outcome for women, newborns, and families. Literature shows that NQMs are a precious entity to healthcare as they are the future of midwifery and hence more research is recommended.

## INTRODUCTION

Newly qualified midwives (NQMs) are valuable individuals as they represent the progression of midwifery^1^. Hence, the ability of these NQMs to effectively make the transition from a student midwife to a registered midwife affects both the midwifery profession and maternity health services^2^. A smooth transition is a key aspect of job retention, especially since many countries worldwide fear the availability of an inadequate number of midwives for future staffing requirements^3^. In the published literature, NQMs describe their transition from student midwives to qualified midwives as a ‘reality shock’^4,5^ which could leave NQMs devastated and unable to process and express their emotions after facing traumatic experiences that could present either ‘too unexpectedly’ or else ‘too soon’, caused by various factors like experiencing abuse from staff members or when facing obstetric emergencies, possibly leading to secondary post-traumatic stress^6^. Such issues highlight the importance of making the transition of NQMs a time that helps them develop confidence and competence^7^.

To qualify as a midwife, one must undergo a midwifery education program that fulfills the academic recognition stipulated by their country, whereby most midwifery courses need to be completed within three to four years to attain a diploma or degree in midwifery studies^8^. Attaining the relevant academic midwifery qualification equips a NQM with the necessary knowledge and skills to care for women during normal pregnancies and assist them in childbirth. Moreover, a NQM is also expected to have the ability to detect complications in both the mother and infant, take preventative measures for these complications and carry out emergency measures for complications that may arise during pregnancy, childbirth, and the postpartum period^9^. An NQM should also have acquired the general knowledge and skills to provide counselling and education on women’s reproductive and sexual health and childcare to women and their families^9^. The international definition of a qualified midwife as stated by the International Confederation of Midwives (ICM)^9^ is: ‘A midwife is a person who has completed a midwifery education program that is based on the ICM Essential Competencies for Basic Midwifery Practice and the framework of the ICM Global Standards for Midwifery Education and is recognized in the country where it is located; who has acquired the requisite qualifications to be registered and/or legally licensed to practice midwifery and use the title ‘midwife’; and who demonstrates competency in the practice of midwifery’^9^.

The experience of childbirth is a particularly important and emotional event in a woman’s life; it will forever affect how she sees herself as a woman, and her relationship with her partner and other family members^10^. This highlights the importance of having NQMs who feel/are confident and competent while caring for women and that a midwife’s well-being has a direct impact on the care provided to a woman^11^. Therefore, an integrative review was undertaken to investigate what NQMs experience as they embark on their new professional role and care for women in the maternity setting.

## METHODS

An integrative review (IR) method combines research and draws inferences from various sources on a topic by looking at and taking into consideration all literature present during the search, being empirical, methodological, and theoretical^12^. As Toronto and Remington^12^ suggest, for both transparency and rigorousness, this review was based on a systematic approach using Cooper’s 1984 framework^12^, which consists of a six-step process that was implemented as guidance. These six steps include: formulating the purpose for the review and formulating a review question; executing a systematic search; selecting appropriate literature; performing a quality appraisal of the literature chosen; carrying out analysis and synthesis of the studies; and discussion, conclusion, and dissemination of the findings^12^.

### Formulating the review questions

The SPIDER method (Table 1) was utilized, this acronym helped to develop the review questions, create keywords, assist in listing both the inclusion and exclusion criteria (Table 2), and guide the literature search^13^. Both authors discussed and agreed on these elements for this IR.

**Table 1:** SPIDER method for developing keywords used for the literature search

*Component*	*Explanation*	*Term*
**Sample**	Types of participants	Newly qualified midwives, newly graduate midwives, junior midwives, midwives on rotation, novice midwives, beginner midwives, trainee midwives, early career midwives, newcomer midwives, apprentice midwives, learner midwives, amateur midwives, newly practicing midwives, newly registered midwives
**Phenomenon of interest**	Area of focus	Labor, birth, intrapartum, parturition, childbirth, different stages of labor, obstetric emergencies, normal vaginal delivery (NVD), confinement, operative birth
**Design**	Methods of data collection	Structured interview, unstructured interview, semi-structured interview, open-ended interview, face-to-face interview, telephone interview, focus group, survey, observation, audio recording, field notes, personal diary, tape-recorded
**Evaluation**	Analysis of experience	Meaning, understanding, lived experiences, views, attitudes, opinions, beliefs, thoughts, perspectives, feelings, perceptions
**Research type**	Methodology of interest	Qualitative, mixed methods, quantitative

**Table 2:** Inclusion and exclusion criteria used to select the studies for this review

*Inclusion*	*Exclusion*
Included keywords in the title or abstract	Described experiences of other newly qualified healthcare professionals such as nurses, doctors, physiotherapists, etc.
Relevant to the review questions	Main aim was assessing only the programs offered to NQMs and not NQMs’ experiences
Explored the experiences of NQMs, including enabling and hindering factors	Only explored student midwives’ experiences and not those of NQMs
Compared senior and student midwives to NQMs experiences	
In the English language	
Any research design: quantitative, qualitative and mixed methods	

Keywords (Table 1) were sought with the help of the medical subject heading (MeSH) database. Consequently, the following three review questions were formulated to help guide the literature search: ‘What are the experiences and feelings of NQMs when caring for women in the maternity setting?’, ‘What may influence the NQM experiences on the job?’, and ‘What strategies may enhance the NQM’s new role?’. Before commencing the literature search, the keywords were combined with the Boolean operators AND and OR, with an asterisk (*) used at the end of certain truncated words to search for all possible endings, and the use of the question mark (?) to replace or represent more than one letter^14^.

### Systematic search and selection of literature

**Figure 1:**
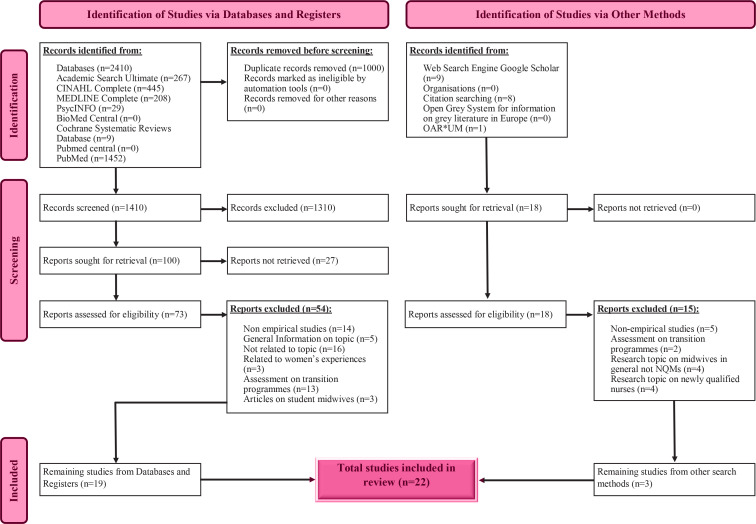
PRISMA flow chart

An extensive literature search was completed by the first author, between December 2021 and the end of July 2022 by combining the keywords presented in Table 1. The following eight databases were used to search the literature: Academic Search Ultimate, Cinahl Complete, Medline Complete, PSYch INFO, BioMed Central, Cochrane Database of Systematic Reviews, PubMed, PubMed Central, and the use of the Google Scholar search engine. All quantitative, qualitative, and mixed-method studies addressing the review questions, were considered for inclusion in this review. No limiters such as the location of keywords, full text, original articles, language, and sample were set during the main search. Limiters were not used since an initial quick literature search indicated that studies published on the topic were few. Hence, to avoid missing any important studies, all that was available was taken into consideration. This was also applicable for the time limit; since no relevant publications dating further back than the year 2000 were found, no time limit was set. Furthermore, the reference lists of the chosen articles were thoroughly scrutinized for any other relevant studies. The retrieved articles were retained according to the inclusion and exclusion criteria (Table 2) for this review. A total of 91 articles were retrieved in full text and, after applying the exclusion criteria, a total of 22 studies were retained for the review. Figure 1 displays the PRISMA (Preferred Reporting Items for Systematic Reviews and Meta-Analyses) flow diagram showing the number of records identified, included, excluded, and the reasons for exclusion^15^.

### Quality appraisal

To determine if the results of the study were valid and could be used for further research studies, education, policies, or clinical practice, an evaluation for utility and quality was done using appraisal tools. Table 3 shows the appraisal tools used for each study included in this review according to its research approach. Table 4 gives a summary of the final 22 studies used in this integrative review, showing the authors, year of publication, methodology, sample size and sampling, the participants, and the data collection methods used. It also indicates the region/country where the study was done, giving a cultural context perspective that could reflect cultural implications in the results. The studies were appraised by the first author, and the processes were reviewed by the second author. The outcomes were discussed and agreed upon by both authors.

**Table 3:** The three tools used to appraise the different research approaches of each study

*Research approach*	*Studies*	*Appraisal tool*
**Qualitative studies**	Avis et al.^29^ Barry et al.^19^ Barry et al.^1^ Cazzini et al.^24^ Clements et al.^2^ Fenwick et al.^34^ Griffiths et al.^21^ Hobbs^22^ Kitson Reynolds et al.^20^ Kool et al.^25^ Naqshbandi et al.^27^ Norris^28^ Saliba^31^ Sheehy et al.^8^ Simane-Netshisaulu and Maputle^33^ Skirton et al.^26^ van der Putten^5^ Wain^4^ Watson and Brown^23^ Young^35^	The Critical Appraisal Program (CASP) qualitative studies tool
**Quantitative studies**	Davis et al.^37^	Critical Appraisal of a Cross-sectional Study (Survey) from the Centre for Evidencebased Management (CEBMa)
**Mixed-method studies**	Lennox et al.^30^	Mixed-Methods Appraisal Tool (MMAT)

**Table 4:** Summary of literature included in the review

*Authors Year*	*Location*	*Methodology*	*Sample size*	*Sample technique*	*Participants*	*Data collection*
Cazzini et al.^24^ 2022	Republic of Ireland	Qualitative study	7	Convenience sampling	NQMs who had commenced their post registration clinical practice in a large teaching hospital	Semi-structured interviews
Simane-Netshisaulu and Maputle^33^ 2021	South Africa	Qualitative study	5	Non probability purposive sampling	NQMs working in selected maternity units during their first year from completion of training	Unstructured in-depth interviews
Sheehy et al.^8^ 2021	Australia	Qualitative study	28	Non probability sampling	Re-recruited participants from a previous longitudinal study	Semi-structured telephone interviews
Watson and Brown^23^ 2021	Northern Ireland	Qualitative study	8	Purposive sampling	NQMs who had obtained their registration in the last 12 months and taken a post with Health and Social Care trust	Semi-structured in-depth interview
Kool et al.^25^ 2020	Netherlands	Qualitative study	21	Snowball sampling	NQMs graduated less than 3 years ago and work as hospital-based midwives in Netherlands	Interviews
Naqshbandi et al.^27^ 2019	Erbil Iraqi	Qualitative study	15	Convenience sampling	NQMs who were in their transition period	Semi-structured in-depth interview
Norris^28^ 2019	United Kingdom	Qualitative study	5	Convenience sample	NQMs in a maternity unit	Focus groups and reflective diary
Griffiths et al.^21^ 2019	Australia Queensland	Qualitative study	8	Purposive sampling	Midwives who had just completed their BMid as part of the RPMEP	Semi-structured telephone interviews
Wain^4^ 2017	United Kingdom	Qualitative study	8	Purposive sampling	NQMs who had completed their preceptorship	Semi-structured interviews
Kitson Reynolds et al.^20^ 2014	United Kingdom	Qualitative study	12	Cohort sampling	NQMs during their first year of practice	Semi-structured interviews
Barry et al.^1^ 2014	Perth Western Australia	Qualitative study	11	Purposive sampling	NQMs who were previously nurses	Interviews and participants journal
Barry et al.^19^ 2013	Perth Western Australia	Qualitative study	11	Purposive sampling	NQMs recruited on their last day as student midwives	Semi-structured interviews and researcher field notes
Clement et al.^2^ 2013	Australia	Qualitative study	38	Purposive sampling	NQMs recruited from 14 public maternity hospitals	Telephone interviews and focus groups
Lennox et al.^30^ 2012	New Zealand	Mixed-methods study	8	Purposive sampling	NQMs who had just been registered as midwives	Semi-structured interviews and telephone logs
Davis et al.^37^ 2012	Australia	Survey	25	Convenience sample	All NQMs employed within three participating Area Health Services	Surveys
Avis et al.^29^ 2012	United Kingdom	Qualitative study	35	Convenience sample	NQMs from 18 work sites	Diary and interviews
Young^35^ 2012	United Kingdom	Qualitative study	36 student midwives 5 midwives 12 mentors	Convenience sampling	Student participants were recruited from the 3-year Preregistration Midwifery	Focus groups, observations and interviews
Hobbs^22^ 2012	United Kingdom	Qualitative study	7	Non-probability sample	NQMs working in the major maternity department	Observations, interviews and personal field diary
Skirton et al.^26^ 2012	UK	Qualitative study	35	Purposive sampling	Final year midwifery students	Diary
Fenwick et al.^34^ 2012	Australia	Qualitative study	16	Convenience sampling	Newly qualified midwives who were participants in a longitudinal project	Interviews
Saliba^31^ 2011	Malta	Qualitative study	11	Purposive sampling	Newly qualified midwives who had just started their employment at Mater Dei Hospital	Interviews
van der Putten^5^ 2008	Ireland	Qualitative study	6	Purposive sampling	Newly qualified midwives who qualified in the last 6 months	Interviews

### Analysis and synthesis

To get a better understanding of the topic, comprehensive methods of data analysis were implemented for this IR, which allowed recasting, combining, reorganizing, and integrating concepts across a body of literature^16^. Data analysis was conducted by deconstructing the articles into their simplest elements^17^. To efficiently analyze the articles, three review matrices were constructed, each representing a review question^12^. The three analysis-matrices used in this review were thoroughly scrutinized for recurrent patterns, and the formation of themes was guided by the review questions^12^. These processes were done by the first author under the supervision of the second author. Both authors discussed and agreed on the findings. Using the Braun and Clarke^18^ six-phase process helped to identify the following three themes: Shocking truth, Beginner’s crisis, and Moving on. This process included familiarizing with data where the article was read multiple times and notes were listed as codes for potential themes; generating initial codes, the data formed from the previous coding was organized into interesting codes; searching for themes, the codes were placed into potential themes and subthemes; reviewing themes where themes were refined by gathering items for each theme and a thematic map was developed (Table 5); defining and naming themes, the aspects of the data each theme captured were determined, and the story of each theme was studied to develop the final theme names; and producing the report where a report of the identified themes was developed.

**Table 5:** Thematic content map showing the process of developing the 1st and 2nd level coding, forming the subthemes and finally the three main themes

*1st Level Coding*	*2nd Level Coding*	*Subthemes*	*Themes*
Learning additional skills Implementing theory to practice Acquired academic/clinical knowledge Staff perception of NQM’s knowledge Lack of experience Self-awareness of skills abilities	Acquired knowledge and experience Abilities and skills	NQMs’ perceptions of what midwifery entails NQMs’ views of the real job experience	**Shocking truth**
Struggles to gain confidence Confidence increases with time More confidence in low-risk situations Job demands and resources Professional demands Competence issues Trust issues Familiarity with work environment Pecking orders Ambivalence Overwhelming emotions Decision-making and accountability Colleagues’ supportive measures Rotation impacting confidence levels Continuity of care helped in confidence Personal demands and resources Increased responsibilities Time management Relationship issues Reality and culture shock Staff attitudes Autonomy Psychological trauma	Confidence variables affecting NQMs’ performance Factors affecting competence levels of NQMs Welcoming work environment Advantages of trust and support of NQMs NQMs in the real working environment Role expectations from students to NQMs Job expectations supersede NQMs’ beliefs Personal coping strategies Hierarchy and relationships Staff/management perceptions of NQMs and their abilities Crisis with increased responsibilities and decision-making Increased thoughts and cognitive processes Feeling and emotions Psychological impact	Experiences and feelings of NQMs Factors affecting NQMs’ role performance	**Beginners’ crisis**
Quitting or adapting Transition phase Training programs Preceptorship Job-satisfaction Supervision Transition programs	Transition and adaptation phase Policies and guidelines Advantages of supervision and training programs Keeping up to date and building on existing knowledge	Coping strategies of NQMs Supervision and support for NQMs	**Moving on**

## RESULTS

### Emergent themes

*Shocking truth*

This theme brings out the stark realities NQMs experience when transitioning from the role of student to that of a registered midwife by looking at NQMs’ perceptions of what midwifery entails and their views of their real job experiences. This theme represents the research question, ‘What may influence the NQM experiences on the job?’. NQMs expressed a feeling of coming into the workforce proud of their graduation and achievements, and embracing the philosophy of midwifery^19^. In one study, this philosophy of midwifery referred to the combination of learned theory, accumulated clinical practice during studentship, and individual experiences of childbirth^19^. NQMs had an idea of what a midwife is, what the job involves, and the relationships they will build with their colleagues, the women they care for, and their families^20-23^. However, this changed over time, once they embarked on the journey of truly becoming a midwife^20^. Participants in many studies experienced a huge surprise when they started practicing in a professional setting, encountering many unexpected scenarios, from what they had been taught and their views on maternity care, to what was practiced in the clinical setting. Leading to a sense of frustration when they could not reach their expectations of their midwifery role^19^.

It emerged that the working environment is based on a medical setting where certain unneeded interventions take place, which led to NQMs looking at the midwifery role as being subordinate^8^. NQMs indicated that the clinical placement could not compare to the midwifery philosophy they believed in^24^. One study particularly showed that the initial feelings of NQMs as they cared for women, were of ‘letting women down’^19^ and not giving them the support, standard of care, and attention, they had envisioned^19,20^. NQMs felt that they were not able to provide proper woman-centered care as they had intended^5,20,21,25,26^, leading to NQMs feeling guilty when they did not dare to speak up for women and stand up to other staff members when they felt that women were not receiving proper care^24^.

However, this could be based on the environment these NQMs were exposed to since in two studies, NQMs had the opportunity to work in caseload midwifery practice. This included student midwives caring for an assigned woman throughout pregnancy, birth, and the postnatal period. This resulted in these NQMs experiencing a completely different aspect of the job description once they were employed as midwives in a hospital-based maternity setting. This is one of the reasons that NQMs became disappointed after having different expectations from the role^20,21^. Whereas NQMs who worked their transition period within the midwifery continuity of care model regarded their experience as a positive one, describing it as having the opportunity to work closely with other midwives, sharing the same philosophy of care, and keeping women at the center of care. In contrast, those from the same study who were assigned to a hospital-based setting expressed negative feedback about their experience^2^.

*Beginners’ crisis*

This theme highlights the difficulties found across the literature that NQMs experience in their new role as registered midwives in a maternity setting. This theme represents the research question: ‘What are the experiences and feelings of NQMs when caring for women in the maternity setting?’. Literature indicates that it is quite common for NQMs to experience a surge of feelings and emotions, which may contribute to a lack of confidence and mediocre performance, making them vulnerable persons during an exceedingly difficult and stressful time of their professional career^8,27,28^. All included studies (n=22) revealed that NQMs experienced some form of stress, with two studies indicating that stress was linked to the phenomenon of sudden status change; from being a protected student to becoming a fully independent and accountable midwife^4,20^. The feeling of losing the shelter provided by their university^20^, stepping into the unknown^29^ and starting to find out the true demands of midwifery was all about experiencing the reality of both sides of the profession, from happy/joyful moments to sad, heartbreaking situations^30^. NQMs revealed that they did not realize the responsibility of being a midwife while they were students and, therefore, found it stressful once they were employed in their new role, taking sole responsibility and decision-making for the women under their care^26^. Three studies show that this sense of responsibility was seen as overwhelming by the participants^5,23,31^.

Studies revealed the steep learning curve that was expected by employers, where NQMs were expected to increase their knowledge regarding certain skills and practices for which they had not been previously trained^8,22^. NQMs were mostly worried about being those who must act during emergencies without any shielding^23^. As students, NQMs never took decisions on their own while caring for women during labor while, once qualified, they were afraid of the heavy responsibility and that they would make a mistake that would cost them their registration^2^. Furthermore, participants put themselves under intense pressure due to increased expectations of their abilities to carry out tasks at the same pace as their senior counterparts^22^. This led to deep frustration, knowing that their ability to transition into their new role would be much slower than they anticipated^32^. Such issues surfaced mostly in birthing units, especially during vaginal examinations (VEs), the second stage of labor, and when interpreting cardiotocography (CTG) results^22,25^.

Participants felt that even though they were educated and trained on responsibility, accountability, and autonomy as students, they did not feel up to these in their first 12 months of employment^20^. NQMs went through so many conflicting ideologies when they tried to use the knowledge they acquired from school into practice, that it resulted in impeding their self-confidence^5,23,27^. Although studies mention that NQMs were conscious of their abilities in knowledge and skills, they mostly felt that their position as an NQM was subordinate, and this unfavorably affected their ability to be sufficiently assertive to deal with the events and tasks presented to them. NQMs described the role of the midwife as intense and emotionally demanding, while not finding the right support from colleagues, which proved to have a negative effect^8,32^. One study compared being accepted by senior midwives to an ‘initiation period’^20^, meaning that they felt as if they had to pass a test to be accepted by their senior counterparts. Other NQMs described their struggle to be accepted by other midwives as a challenge that had to be endured^28^. NQMs felt continuously watched by colleagues, waiting to be judged on whether they stepped out of line and whether they can be trusted^2,21^. They felt that they were mostly seen as a burden, lacking skills, ability, and competence^8,21,23,24,30^. Studies by Kool et al.^25^, Norris^28^and Wain^4^ have all described the dilemmas NQMs face when they need to ask for help from other midwives or refer women to obstetricians. Fearing that they would be seen as weak and incompetent, NQMs felt like a nuisance to ask for help/support, especially in busy environments and short-staffed circumstances, shattering their hopes of building a trusting relationship with colleagues^24,26^. Sheehy et al.^8^ and Simane-Netshisaulu and Maputle^33^ describe a bullying culture where NQMs feel belittled by a hierarchical system and autocratic personalities. This is also shown in the studies of Fenwick et al.^34^ and Lennox et al.^30^ where a pattern of inappropriate working culture was noted in some units, where midwives rank themselves according to seniority with NQMs placed at the bottom of the hierarchy, the well known ‘pecking order’^30^.

Findings of one study revealed how participants felt helpless and did not know what to do to perform their role, with one of the participants expressing that she felt ‘like a fraud’^30^ while speaking to a mother who was continuously thinking that this woman should be speaking to ‘a real midwife’^30^ . On the other hand, in another study, a common expression by NQMs was ‘fake it till you make it’^8^, which involved mimicking skills from other colleagues even though they were not aware of what they were doing^8^.

Most NQMs take all these fears, feelings, and emotions back to their own homes, worrying that they failed as midwives and believing that they did something wrong while caring for women^20,25^. Worst of all, they even compared themselves to others, which, eventually, may take a toll on their personal lives. Participants complained of not being able to sleep and constantly counting the days until their next duty^28^. Others commented on not taking any breaks during their duty or staying on after their duty ended, to see the outcome of the birth even though they were exhausted and needed to go home to their families^22,23^. Their coping skills and anxiety were based on how and if they would manage to complete the tasks assigned to them^28^. Such findings also emerged from a study^22^ focusing on Bourdieu’s notion of habitus, which is defined as ‘our overall orientation to or way of being in the world; our predisposed ways of thinking, acting, and moving in and through the social environment’^22^ . Hence, Hobbs^22^ explains that NQMs tend to overlook their wellbeing since midwifery is a female-dominated profession and it is in their nature to give their 100% and more, which Bourdieu refers to as ‘service and sacrifice’^22^.

Six of the studies included in this review^2,4,8,25,29,34^ presented the experiences of NQMs when working on a rotation basis. This brought about instability in their confidence^8^, insecurity, and having to prove themselves to colleagues in an extremely limited period^24,25^. Time is needed in one clinical area to consolidate and gain knowledge and experience^4^. However, some conflicting ideas emerged between participants, such as in the study by Avis et al.^29^ where some participants agreed with the negative effects of the rotation system, describing it as a ‘roller coaster ride’, while others described it as building their confidence since they had the opportunity to witness various scenarios from different ward settings. Similarly, in Clements et al.^2^, participants working in the continuity of midwifery care setting did not experience any feelings of stress, fear, or anxiety during their rotation. However, the findings of these studies could have been affected by organizational structures and dimensions, the geolocation of the studies, and personal character traits. Workload and time constraints were other factors that made it extremely difficult and stressful for NQMs to provide proper care and attention to women during labour^25^. Moreover, the inability to provide proper woman-centered care was yet another added concern for NQMs when assigned to shifts, as it made them feel that they lacked continuity of care^21^. However, these participants had been given their midwifery training in a caseload midwifery setting, which differs from a hospital-based maternity setting, and hence, their feelings might have been accentuated by this fact^21^.

*Moving on*

The final theme in this review investigates several factors that NQMs identified as helping them accept, adapt, and move forward in their new role, representing the final research question: ‘What strategies may enhance the NQM in her new role?’. Over time, participants started to feel more confident, obtained a clearer picture of what was expected of them^28^ and were able to put their knowledge into practice^26,31^. One study explains that NQMs go through three stages while transitioning from students to qualified midwives to provide woman-centered care^1^. These three stages include: being centered on developing their personal qualities, developing an understanding of outside influences that may impact their practice, and moving through a process to support change through their plan of action^1^. The feeling of belonging to the profession and their role as a midwife helped NQMs not only to provide the best care to mothers but also set boundaries to help themselves survive. These included working autonomously, gaining professional recognition, respecting their family commitments, and supporting their emotional demands^1^. Other factors that encouraged NQMs to retain their profession, and achieve job satisfaction and motivation were the ability to work within the full scope of midwifery practice and building a good relationship with the women in their care^8,25^. Receiving positive feedback and trust from the women they cared for was considered an important job resource, which instilled a feeling of empowerment in new midwives^19^.

Another factor that helped NQMs cope with their transition, was being assigned to work in clinical areas where they previously trained as students helping them to adapt faster in their role due to the familiarity with the ward setting and workforce^8,24,31^. It also helped them in the steep learning curve and to provide better care to women^8,24,31^.

Additionally, two studies point out that the structure of clinical placements previously familiar to student midwives directly influences NQMs once they start to work^21,26^. Hence, being oriented with the clinical setting and equipment^30^, whilst being informed about ward/hospital protocols and guidelines, gave NQMs a feeling of security, knowing what is expected of them in the early days of their employment^8,29^. Likewise, organizational structures within the hospital helped NQMs to work to the full scope of the midwifery practice^8^.

Five studies show that when NQMs were supervised or assigned to a senior midwife, facilitator or preceptor during duties, NQMs’ confidence increased, giving them a sense of security^4,24,25,29,31^ emphasizing that being surrounded by support from colleagues was deemed crucial and helpful^23-25,29,31^. Naqshbandi et al.^27^ add that supportive colleagues help NQMs to adapt faster, have a smooth transition, increase confidence, and make them more likely to retain their profession. This support offered NQMs positive learning experiences in practice^8,28^ and helped with stress and anxiety^24^. Fitting in and feeling accepted and not judged was an inspirational goal for NQMs^28,34^. NQMs also expressed that being in the company of colleagues during duties and breaks, team building activities and socializing during out of duty hours were all assets in building relationships and enhancing their work practice^8,24,25^.

NQMs’ decision-making skills were developed when they were assigned to another colleague and were given the possibility to speak about their concerns^35^. Moreover, decision-making skills were also improved when senior midwives dedicated time to explain and discuss with NQMs the rationale behind a decision rather than leaving them to fend on their own^35^ or taking over their work^30^. Senior midwives must become aware that their positive support encourages NQMs to remain in employment^27^, especially with the increasing concern of the availability of an insufficient number of midwives for future staffing^3^.

Apart from being supported, debriefing was also found to be vital for NQMs to survive and move on in their midwifery role^8^. Participants explained that having the space to vent their feelings about their experiences and challenges, helped them to relieve their anxiety, learn, and progress^8,30^. Furthermore, having a friend who is going through the same experience also helped in relieving anxiety^23,24^. Additionally, having the possibility of assigning NQMs to a one-to-one woman-care ratio during the first few months of work is deemed to be a beneficial consideration^4^. Being placed in a shift that is made up of mixed levels of staffing experience^8^ and is considered supernumerary^23,24^ was also seen as an important strategy for learning and safety in practice.

Another factor found to enhance the transition phase of NQMs was having good organizational support, which was shown to be an asset for NQMs. This support translates to having sound induction programs allowing NQMs to orient themselves with units and equipment^30^, be introduced to policies and guidelines^8,29^, be assigned to preceptors, have supervision, and be assigned over and above the staff requirement of the clinical area^8,23^. These provided NQMs with a smooth transition into the workforce. The organization should endeavor to support and empower NQMs to further their studies to allow them to expand their knowledge, and stay up-to-date with the latest evidence-based literature, besides keeping them motivated and giving them a sense of belonging^25^. Feeling appreciated and being acknowledged by the system as a contributing professional was perceived positively by NQMs^8^.

## DISCUSSION

This review aimed to explore the experiences of NQMs when caring for women in the maternity setting. The literature demonstrated how NQMs’ wellbeing is at stake after registration, as they go through a tumultuous period full of psychological stress, fear, and other surges of negative emotions since the role of the midwife is very intense and emotionally demanding^8^. This difficult period is linked to the new responsibilities of their role, especially when it comes to decision-making, which may influence their confidence and competency^20^. The sudden change of role to a more demanding and responsible one might come to what was explained by Kramer in 1974^4,5^, as a ‘reality shock’, where what was expected and envisioned as students becomes shattered once they join the workforce^20-22^. Participants felt helpless in the early days of their professional role as they did not have the understanding or the experience to perform their roles effectively and efficiently^30^. Feelings of incompetence and lack of self-confidence had an increased influence on the stress levels of NQMs, causing a psychological impact on them since they had become the sole-accountable person for the mother and baby under their care. Most were afraid to assume this newly acquired responsibility out of fear of making a serious mistake, which would cost them their midwifery licence^2^. All these emotions, together with lofty expectations and the lack of support from colleagues, were taking a toll on their personal lives, which could lead to compassion fatigue, which is known as the ‘cost of caring’^6^, especially when exposed to obstetric emergencies, leading to less compassion satisfaction^36^. These emotions were mostly dependent on the different settings NQMs were enrolled in since participants felt less psychologically affected and more at ease when they were not assigned to birthing units in a hospital-based setting but worked in caseload midwifery practice^2^.

Most of the literature reviewed also highlights the transition phase from being a student to becoming a midwife^1,2,4,8,19-25,27,28,30,31,34,35,37^. The findings from the studies concur with a three-phase gradual transition where, primarily, NQMs felt the loss of what was familiar to them; teachers, school colleagues, mentors, and the shelter provided by their university^20^, whilst stepping into a whole new world and a role of uncertainty^29^. This IR also showed the importance for NQMs to form new and trusting relationships with colleagues and women patients, trying to fit in and carry out the necessary skills needed to perform tasks. Finally, it became evident that as time passed, participants were finally reaching the stage where they wanted to be, in the ‘promised land’^28^ . In this new phase of adaptation, although still scary and risky, NQMs now feel they have a clearer vision of what is expected of them^28^ and start to apply all the knowledge they already possess with that they continue to acquire from their new experiences^1^. They are ready to let go of what passed and try to adapt to their new role and environment as more autonomous midwives^28^, leading to job satisfaction where they feel they are finally truly helping women^8,25^. Senior midwives were found to play one of the biggest roles in guiding and helping NQMs succeed^25,29,31^. When senior midwives were readily available to explain, guide, and help without any form of judgment or exercise of superiority, this was the ideal situation for NQMs to learn and advance in their practice^2,21,25,27,29,31^. Kitson Reynolds^20^ and Cazzini et al.^24^ emphasized the importance of giving NQMs time and space for debriefing and considered this to be instrumental for all healthcare professionals. Debriefing should not only be available after an emergency or traumatic experience but for anyone who needs it^20^.

## CONCLUSIONS

Transitioning from a student to a midwife brings about stress and tension, especially as NQMs take full responsibility for the women under their care, knowing that their decisions can have a direct impact on the outcome for women, newborns, and families. Literature shows that the factors that influence NQMs include the working environment, especially when working in birthing units, the support given by colleagues, and the organization in which they are employed. It is therefore very important that NQMs find the needed support to be able to overcome this trauma before it is aggravated and leads to secondary post-traumatic stress. NQMs are a precious entity in healthcare, as they are the future of midwifery. Yet, research is still extremely limited, and, to date, the focus has mostly been on the transition to practice and on supportive programs such as preceptorship. Further research focusing on more holistic experiences of NQMs is warranted to enable a deeper understanding of their professional development.

## Data Availability

Data sharing is not applicable to this article as no new data were created.

## References

[1] Barry MJ, Hauck YL, O’Donoghue T, Clarke S (2014). Newly-graduated midwives transcending barriers: mechanisms for putting plans into actions. Midwifery.

[2] Clements V, Davis D, Fenwick J (2013). Continuity of Care: Supporting New Graduates to Grow Into Confident Practitioners. Int J Childbirth.

[3] World Health Organization (2016). Global strategic directions for strengthening nursing and midwifery 2016-2020.

[4] Wain A (2017). Examining the lived experiences of newly qualified midwives during their preceptorship. Br J Midwifery.

[5] van der Putten D (2008). The lived experience of newly qualified midwives: A qualitative study. Br J Midwifery.

[6] Missouridou E (2017). Secondary Posttraumatic Stress and Nurses’ Emotional Responses to Patient’s Trauma. J Trauma Nurs.

[7] Rahmadhena MP, McIntyre M, McLelland G (2017). New midwives’ experiences of transition support during their first year of practice: a qualitative systematic review protocol. JBI Database System Rev Implement Rep.

[8] Sheehy A, Smith R, Gray J, Ao CH (2021). Understanding workforce experiences in the early career period of Australian midwives: insights into factors which strengthen job satisfaction. Midwifery.

[9] International Confederation of Midwives International Definition of the Midwife.

[10] Humenick SS (2006). The Life-Changing Significance of Normal Birth. J Perinat Educ.

[11] Beaumont E, Durkin M, Hollins Martin CJ, Carson J (2016). Compassion for others, self-compassion, quality of life and mental well-being measures and their association with compassion fatigue and burnout in student midwives: A quantitative survey. Midwifery.

[12] Toronto CE, Remington R (2020). A Step-by-Step Guide to Conducting an Integrative Review.

[13] Dibley L, Dickerson S, Duffy M, Vandermause R (2020). Doing Hermeneutic Phenomenological Research: A Practical Guide.

[14] Ecker ED, Skelly AC (2010). Conducting a winning literature search. Evid Based Spine Care J.

[15] Page MJ, McKenzie JE, Bossuyt PM (2021). The PRISMA 2020 statement: An updated guideline for reporting systematic reviews. Int J Surg.

[16] Torraco RJ (2016). Writing Integrative Literature Reviews: Using the Past and Present to Explore the Future. Human Resource Development Review.

[17] Torraco RJ (2005). Writing Integrative Literature Reviews: Guidelines and Examples. Human Resource Development Review.

[18] Braun V, Clarke V (2006). Using thematic analysis in psychology. Qual Res Psychol.

[19] Barry MJ, Hauck YL, O’Donoghue T, Clarke S (2013). Newly-graduated midwives transcending barriers: a grounded theory study. Midwifery.

[20] Kriston Reynolds E, Cluett E, Le-May A (2014). Fairy tale midwifery—fact or fiction: The lived experiences of newly qualified midwives. Br J Midwifery.

[21] Griffiths M, Fenwick J, Carter AG, Sidebotham M, Gamble J (2019). Midwives transition to practice: Expectations and experiences. Nurse Educ Pract.

[22] Hobbs JA (2012). Newly qualified midwives’ transition to qualified status and role: Αssimilating the ‘habitus’ or reshaping it?. Midwifery.

[23] Watson H, Brown D (2021). Experiences of newly qualified midwives working in clinical practice during their transition period. Br J Midwifery.

[24] Cazzini H, Cowman T, Fleming J (2022). An exploration of midwives’ experiences of the transition to practice in the Republic of Ireland. Br J Midwifery.

[25] Kool LE, Schellevis FG, Jaarsma DADC, Feijen-De Jong EI (2020). The initiation of Dutch newly qualified hospital-based midwives in practice, a qualitative study. Midwifery.

[26] Skirton H, Stephen N, Doris F, Cooper M, Avis M, Fraser DM (2012). Preparedness of newly qualified midwives to deliver clinical care: An evaluation of pre-registration midwifery education through an analysis of key events. Midwifery.

[27] Naqshbandi PS, Karim MA, Qadir DO (2019). A qualitative Study of the Recent Midwifery Graduates’ lived Experiences During Transition Period. International Journal of Medical Investigation.

[28] Norris S (2019). In the wilderness: an action-research study to explore the transition from student to newly qualified midwife. Evidence Based Midwifery.

[29] Avis M, Mallik M, Fraser DM (2013). ‘Practising under your own Pin’– a description of the transition experiences of newly qualified midwives. J Nurs Manag.

[30] Lennox S, Jutel A, Foureur M (2012). The Concerns of Competent Novices during a Mentoring Year. Nurs Res Pract.

[31] Saliba A (2011). Becoming a midwife in Malta : a single case study. Dissertation.

[32] Benner P (1984). FROM NOVICE TO EXPERT EXCELLENCE AND POWER IN CLINICAL NURSING PRACTICE. American Journal of Nursing.

[33] Simane-Netshisaulu KG, Maputle MS (2021). Exploring supportive relationship provided to newly qualified midwives during transition period in Limpopo province, South Africa. Afr J Reprod Health.

[34] Fenwick J, Hammond A, Raymond J (2012). Surviving, not thriving: a qualitative study of newly qualified midwives’ experience of their transition to practice. J Clin Nurs.

[35] Young N (2012). An exploration of clinical decision-making among students and newly qualified midwives. Midwifery.

[36] Katsantoni K, Zartaloudi A, Papageorgiou D, Drakopoulou M, Misouridou E (2019). Prevalence of Compassion Fatigue, Burn-Out and Compassion Satisfaction Among Maternity and Gynecology Care Providers in Greece. Mater Sociomed.

[37] Davis D, Foureur M, Clements V, Brodie P, Herbison P (2012). The self reported confidence of newly graduated midwives before and after their first year of practice in Sydney, Australia. Women Birth.

